# Patterns of antipsychotic prescription and accelerometer-based physical activity levels in people with schizophrenia spectrum disorders: a multicenter, prospective study

**DOI:** 10.1097/YIC.0000000000000433

**Published:** 2022-09-15

**Authors:** Vincenzo Oliva, Giuseppe Fanelli, Manuel Zamparini, Cristina Zarbo, Matteo Rocchetti, Letizia Casiraghi, Fabrizio Starace, Alessandra Martinelli, Alessandro Serretti, Giovanni de Girolamo, the DiAPASon Consortium

**Affiliations:** a Department of Biomedical and Neuromotor Sciences, University of Bologna, Bologna, Italy; b Department of Human Genetics, Radboud University Medical Center, Donders Institute for Brain, Cognition and Behaviour, Nijmegen, the Netherlands; c Unit of Epidemiological and Evaluation Psychiatry, IRCCS Istituto Centro San Giovanni di Dio Fatebenefratelli, Brescia; d Department of Mental Health and Dependence, ASST of Pavia; e Department of Brain and Behavioral Sciences, University of Pavia, Pavia; f Department of Mental Health and Dependence, AUSL of Modena, Modena; g Department of Neurosciences, Biomedicine and Movement Sciences, University of Verona, Verona; h Unit of Clinical Psychiatry, IRCCS Istituto Centro San Giovanni di Dio Fatebenefratelli, Brescia, Italy

**Keywords:** actigraph, antipsychotics, monopharmacy, physical fitness, polypharmacy, psychiatric residential treatment facilities, psychosis, psychotropic drugs, rehabilitation, wearable accelerometer-based biosensor

## Abstract

Antipsychotic polypharmacy (APP) in patients with schizophrenia spectrum disorders (SSDs) is usually not recommended, though it is very common in clinical practice. Both APP and SSDs have been linked to worse health outcomes and decreased levels of physical activity, which in turn is an important risk factor for cardiovascular diseases and premature mortality. This real-world, observational study aimed to investigate antipsychotic prescribing patterns and physical activity in residential patients and outpatients with SSDs. A total of 620 patients and 114 healthy controls were recruited in 37 centers across Italy. Each participant underwent a comprehensive sociodemographic and clinical evaluation. Physical activity was monitored for seven consecutive days through accelerometer-based biosensors. High rates of APP were found in all patients, with residential patients receiving more APP than outpatients, probably because of greater psychopathological severity. Physical activity was lower in patients compared to controls. However, patients on APP showed trends of reduced sedentariness and higher levels of light physical activity than those in monopharmacy. Rehabilitation efforts in psychiatric residential treatment facilities were likely to result in improved physical activity performances in residential patients. Our findings may have important public health implications, as they indicate the importance of reducing APP and encouraging physical activity.

## Introduction

Many drug utilization studies have found significant variability in prescribing patterns of psychotropic drugs and have highlighted high rates of polypharmacy (de Girolamo *et al.*, 1987; Gallego *et al.*, 2012). High frequencies of polypharmacy and off-label prescriptions have been consistently found in patients in treatment at mental health services in Italy (Bellantuono *et al.*, 1981; Muscettola *et al.*, 1987; Muscettola *et al.*, 1991; Carton *et al.*, 2015). In particular, antipsychotic polypharmacy (APP) is prescribed to a substantial proportion of patients suffering from severe mental disorders, approaching 23% in Europe (Gallego *et al.*, 2012). Up to 44.4% of patients with a schizophrenia spectrum disorder (SSD) receive a combination of antipsychotics, whereas at least 24.4% receive three or more antipsychotics (Fisher *et al.*, 2014). However, the clinical utility of APP is often debated. In selected clinical conditions, including treatment-resistant schizophrenia, antipsychotic-induced hyperprolactinemia and metabolic disturbances in patients receiving clozapine, APP may be indicated (Correll *et al.*, 2009; Esteves *et al.*, 2015; Cooper *et al.*, 2016; De Berardis *et al.*, 2020; Caliskan *et al.*, 2021). Nevertheless, the increased risk of adverse events, drug-drug interactions, decreased adherence to complex drug regimens and consequent risk of early relapse, as well as the higher costs associated with polypharmacy should lead clinicians to exercise caution when using multiple medications at the same time, particularly in the case of long-term treatments (Galling *et al.*, 2017; Gundogmus *et al.*, 2021). The use of APP in clinical practice may sometimes be justified also by the desire to improve the therapeutic response (Lahteenvuo and Tiihonen, 2021). However, even in this case, it is known that particular care must be taken when combining a partial dopaminergic agonist and a full D2 receptor antagonist, which may increase the risk of clinical relapse or worsening of psychotic symptoms (Lippi *et al.*, 2022). The relevant gap between evidence and routine practice, therefore, needs to be further investigated. Of particular interest are the prescription patterns in psychiatric residential treatment facilities, where previous studies have shown that polypharmacy was common, with an average of 2.7 drugs prescribed for each treated patient (Tomasi *et al.*, 2006). It is necessary to understand to which extent different treatment settings (outpatient care vs. residential care) are associated with different prescription patterns.

Several studies have also indicated that both severe psychoses and antipsychotic use are associated with lower levels of physical activity and consequently lower levels of physical fitness. Indeed, a meta-analysis has shown that people with severe mental disorders were significantly more sedentary and less likely to meet physical activity targets established by international guidelines than healthy controls (Vancampfort *et al.*, 2017). Even at a young age, patients taking antipsychotic drugs are less physically active and have a compromised body balance compared to adolescents not treated with antipsychotics (Vancampfort *et al.*, 2016), and illness chronicity has been identified as a worsening factor in physical activity (Walther *et al.*, 2015). Low levels of physical activity and a lower physical fitness are important risk factors for cardiovascular diseases and premature mortality (Kodama *et al.*, 2009), and this becomes even more relevant in patients with psychosis, who have higher standardized mortality rates compared to the general population (Simon *et al.*, 2018). Physical activity may help reduce the risk of weight gain and metabolic syndrome, as well as tobacco and substance use (Mittal *et al.*, 2017), which are often observed in people with schizophrenia or treated with antipsychotics. Furthermore, it is well known that physical exercise can improve cognitive functioning and facilitate neurogenesis in areas of the brain affected by psychosis (Firth *et al.*, 2017). For this reason, it is important to explore physical activity levels in patients with SSDs to enable targeted and preventive interventions. The largest part of previous studies investigating the relationship between severe mental disorders and physical activity was based on retrospective physical activity self-reports. Although such measures are related to a series of advantages (e.g. limited costs and ease of implementation across a large variety of populations and settings), physical activity tends to be misreported, with much higher or lower amounts of activity being recalled than those reported in studies using objective measures (Prince *et al.*, 2008). The inaccuracy of self-reports may be even more exaggerated in people with SSDs because of particularly common recall errors (Firth *et al.*, 2018). The recent availability of wearable devices allows real-time detection of physical activity and may much improve knowledge and management of rehabilitation programs. Accelerometers are small and non-invasive, and they can measure physical activity by quantifying movement with sampling frequencies that can reach 100 observations per second (Hz) while providing an objective assessment of movement-based physical activity across the entire intensity spectrum (from zero to maximal exertion). The close investigation of physical activity using objective measures can allow the collection of valuable data to study the relationship between antipsychotic prescribing patterns and physical activity (Chen *et al.*, 2016; Wee *et al.*, 2019).

The present study will therefore investigate antipsychotic prescribing patterns and physical activity measurements conducted with wearable accelerometer-based biosensors in a sample of people with SSDs.

## Materials and methods

From October 2020 to October 2021, 620 patients (i.e. 313 residential patients (RPs) and 307 outpatients) with a diagnosis of an SSD were recruited in 37 Departments of Mental Health (DMH) or psychiatric residential treatment facilities across Italy as part of the DAily time use, Physical Activity, quality of care and interpersonal relationships in patients with Schizophrenia spectrum disorders (DiAPASon) project (de Girolamo *et al.*, 2020). We included patients with a Diagnostic and Statistical Manual of mental disorders (DSM)-5-based diagnosis of any SSDs (American Psychiatric Association, 2013) who were 20–55 years old and able to speak and write in Italian. We excluded patients who were unable to provide informed consent or who reported severe cognitive deficits (i.e. a Mini-Mental State Examination corrected score lower than 24), a recent diagnosis of substance use disorder according to DSM-5 criteria, a history of clinically significant head injury or cerebrovascular/neurological disease. In each study center, clinicians invited their patients to enter the study. Participants were provided with detailed information about the study and had the opportunity to ask questions. Some of the assessment tools were administered by the treating clinician, whereas research assistants helped patients to complete self-reported questionnaires.

In the same period, 114 healthy controls were recruited from the general population through advertisements, both on the project website and social networks, to take part in the actigraphy study. The healthy controls had no history of psychiatric disorders according to DSM-5 criteria and were excluded based on the same criteria used for the recruitment of patients. The healthy controls were paired for age and sex with the clinical sample who performed the accelerometer-based physical activity measurement.

The accelerometer-based physical activity measurement was undertaken in 10 of the participating centers due to organizational and logistic problems which prevented the implementation of the biosensor study in the remaining study sites. The monitoring was preceded by a briefing session in which the research assistant gave instructions about the procedures and how to effectively perform them and was followed by a debriefing section in which the same research assistant collected information on study acceptability and feasibility. During the debriefing session, outpatients and healthy controls received € 25,00 for travel expense reimbursement.

The DiAPASon study has been approved by the ethical committees of the three main participating centers, which are, IRCCS Istituto Centro San Giovanni di Dio Fatebenefratelli (31/07/2019; no. 211/2019), Area Vasta Emilia Nord (25/ 09/2019; no. 0025975/19), Pavia (02/09/2019, no. 20190075685), and by the ethical committees of all the other participating sites.

### Assessment of clinical variables

For each recruited patient, we completed a sociodemographic and clinical assessment, and several validated clinical scales were administered; for details see the study protocol (de Girolamo *et al.*, 2020). The 24-item Brief Psychiatric Rating Scale (BPRS) (Overall and Gorham, 1962; Morosini and Casacchia, 1995) was used to assess the presence and severity of psychopathology; BPRS items were rated on a seven-point scale ranging from 1 (not present) to 7 (extremely severe). Negative symptoms severity was assessed using the Brief Negative Symptom Scale (BNSS) (Strauss *et al.*, 2012; Mucci *et al.*, 2015), a 13-item instrument designed for the evaluation of blunted affect, alogia, asociality, anhedonia and avolition (from 0 – not present to 6 – severe deficit). For both BPRS and BNSS, higher total scores denote higher severity of symptomatology. The 43-item Specific Levels of Functioning Scale (SLOF) (Montemagni *et al.*, 2015) was used for the assessment of psychosocial functioning. The SLOF is a multidimensional behavioral survey comprising six subscales: physical functioning, personal care skills, interpersonal relationships, social acceptability, activities of community living, and work skills. The SLOF items were rated on a five-point scale ranging from 1 to 5. Higher scores denote the higher functioning of the patient. The self-reported WHO Disability Assessment Schedule (WHODAS) 2.0 (Federici *et al.*, 2009; Gold, 2014) was used to assess disability across six functional domains: that is, cognition, mobility, self-care, getting along, life activities and participation. The items of the WHODAS 2.0 range from 0 to 4, and higher scores indicate a higher functional disability. The Charlson Comorbidity Index (CCI) (Charlson *et al.*, 1987) was used to assess the somatic comorbidity of all participants. The CCI consisted of 19 items corresponding to different medical comorbid conditions; the total score consists of the sum of the conditions presented, with higher scores indicating more severe comorbid conditions.

### Assessment of physical activity

Physical activity was monitored through the multisensor device Actigraph GT9X Link, which is a validated triaxial accelerometer that includes a gyroscope, magnetometer, secondary accelerometer and Bluetooth capability manufactured by ActiGraph, LLC (https://actigraphcorp.com/actigraph-link/). The Actigraph GT9X provides reliable data about metabolic equivalents, activity intensity and sleep efficiency/quality. The Actigraph was worn on the non-dominant wrist for seven consecutive days.

### Data management

Upon the return of both devices, data were uploaded using ActiLife (Actigraph, Pensacola, Florida, USA) and saved in raw format as GT3X+ files. Individual ActiGraph’s.gt3x files were processed using the GGIR R package (Migueles *et al.*, 2019) (with default settings). To estimate the Euclidean norm of the acceleration in x/y/z axes and to separate out the activity-related component of the acceleration signal, we removed one gravitational unit from the vector magnitude (with remaining negative values truncated to zero) obtaining Euclidean Norm Minus One (ENMO). To describe the overall level and distribution of physical activity intensity, we combined the sample level data into 60 s epochs for summary data analysis, maintaining the average vector magnitude value over the epoch. To represent the distribution of time spent by an individual in different levels of physical activity intensity, we generated an empirical cumulative distribution function from all available 60 s epochs. Non-wearing epochs, defined as stationary periods, were estimated using a 60 min window and the default GGIR algorithm and removed (no data imputation was performed) (van Hees *et al.*, 2013). A valid day was defined as having at least 10 h of wearing time, and a valid subject was defined as having at least four valid days.

For each epoch, oxygen consumption (VO_2_) was estimated through the formula:


$$\begin{array}{l} VO_{2}=0.901\cdot ENMO^{0.534} \end{array}$$

If VO_2_ was less than 3.0, we set to floor of 3.0 and computed the metabolic equivalent of task (MET) as:


$$\begin{array}{l} MET=VO_{2}/3.5 \end{array}$$

One MET is defined as the energy used when resting or sitting still (i.e. an activity that has a value of four METs means that the subject is consuming four times the energy than would if he/she was sitting still).

To get a categorial measure of physical activity intensity in each epoch, we finally categorized the number of METs as follows (Hildebrand *et al.*, 2014):

(1) METs ≤1.5 = sedentary;
(2) 1.5<METs<3.0 = light;
(3) 3.0≤METs<6.0 = moderate;
(4) METs ≥6.0 = vigorous.

### Statistical analyses

Frequencies and percentages for categorical variables and means and SDs for continuous variables were computed. Chi-squared or Fisher’s exact tests were used according to the nature of the data to compare categorical variables between groups. The distribution of continuous variables was established using Kolmogorov–Smirnov normality tests. *T*-tests and ANOVA, or the nonparametric Mann−Whitney and Kruskal–Wallis tests, were used for continuous variables as appropriate. Bonferroni post hoc tests were also performed to identify which pairs of means were statistically different when ANOVA or Kruskal–Wallis tests were significant. Effect sizes were estimated with Phi coefficient for categorical variables and Cohen’s d (standardized mean difference) for continuous variables. All analyses were carried out using SPSS software (IBM, Version 27.0) and SAS Studio (SAS Institute Inc. 2015), with the statistical significance level set at 0.05, given the exploratory nature of this study.

## Results

### Sociodemographic and clinical characteristics of the sample

The sociodemographic and clinical features of the sample are reported in detail in Table 1. Patients with SSDs and healthy controls were comparable with respect to age but differed with respect to the other sociodemographic variables considered. In particular, controls included more female subjects, they were more likely to cohabit, have higher education, and be involved in work activities than patients.

**Table 1:** Sociodemographic and clinical features of patients (on antipsychotic mono- and polypharmacy) and healthy controls

Variables	Patients on antipsychotic monopharmacy *N* = 316 (52.1%)	Patients on antipsychotic polypharmacy *N* = 291 (47.9%)	*χ*^2^/U	Effect size	*P* value^a^	Healthy controls *N* = 114
Sex, *N* (%)						
Mal*e*	215 (68.0%)	202 (69.4%)	0.134	0.015	0.715	66 (57.9%)^b^
Age (mean, SD)	41.2 (9.5)	41.7 (9.3)	89.763	0.053	0.547	41.6 (10.3)
Marital status, *N* (%)						
Single	259(82.0%)	266 (91.4%)	16.086	0.163	**<0.001**	30 (26.3%)^b^
Married or cohabiting	34 (10.8%)	8 (2.8%)				77 (67.5%)^b^
Divorced or widowed	23 (7.3%)	17 (5.8%)				7 (6.1%)^b^
Education (mean, SD)	11.9 (3.1)	11.4 (3.1)	84.345	0.161	0.052	16.6 (4.9)^b^
Working status, *N* (%)						
Working	65 (20.6%)	58 (19.9%)	0.041	0.008	0.980	104 (91.2%)^b^
Studying	18 (5.7%)	17 (5.8%)				8 (7.0%)^b^
Not working/studying	233 (73.7%)	216 (74.2%)				2 (1.8%)^b^
BMI (mean, SD)	27.9 (5.4)	27.6 (5.6)	86.029	0.054	0.259	24.4 (4.0)^b^
Waist circumference (mean, SD)	100.8 (20.5)	99.8 (23.5)	87.601	0.045	0.689	88.8 (12.6)^b^
Smokers, *N* (%)	155 (49.1%)	175 (60.6%)	8.056	0.115	**0.005**	NA
Smoking (cigarettes per day) (mean, SD)	7.8 (10.0)	10.4 (11.0)	94.956	0.247	**0.001**	NA
CCI (mean, SD)	0.7 (1.1)	0.8 (1.3)	89.895	0.083	0.452	NA
Illnessduration(mean, SD)	17.2 (9.3)	19.5 (9.6)	94.643	0.243	**0.004**	NA
Lifetime duration of psychiatric hospitalizations, *N* (%)						
<1 year	176 (55.7%)	108 (37.1%)	31.041	0.226	**<0.001**	NA
1–5 years	84 (26.6%)	77 (26.5%)				NA
>5 years	56 (17.7%)	106 (36.4%)				NA
BPRS (mean, SD)	43.8 (12.9)	49.6 (16.9)	97.526	0.386	**<0.001**	NA
BNSS (mean, SD)	20.9 (14.9)	24.9 (16.3)	95.191	0.256	**0.002**	NA
SLOF (mean, SD)	181.8 (22.2)	169.6 (36.0)	77.892	0.408	**<0.001**	NA
WHODAS 2.0 (mean, SD)	12.6 (9.1)	13.2 (9.6)	90.007	0.064	0.475	NA

a Chi-square test for categorical variables, Mann–Whitney-U test for continuous variables.

b For these variables, healthy controls are significantly (*P* < 0.05) different from patients.

BPRS, Brief Psychiatric Rating Scale; BNSS, Brief Negative Symptom Scale; CCI, Charlson Comorbidity Index; N, number; SLOF, Specific Levels of Functioning Scale; WHODAS 2.0, WHO Disability Assessment Schedule.

Looking at the patient groups, patients on APP were more likely to have no partners, had a longer duration of illness and spent more time in psychiatric hospitalizations than patients on antipsychotic monopharmacy (APM). Furthermore, significant differences were found in BPRS and BNSS scores between patients on APM and APP, with the latter showing higher psychopathologic and negative symptoms severity. Psychosocial functioning, as measured by SLOF, was significantly better in patients on APM than on APP. Concerning patients’ physical health, patients on APM and APP did not differ in terms of BMI, waist circumference and CCI. Regarding smoking habits, patients on APP were more likely smokers than those on APM, with a great number of cigarettes per day smoked.

Treatment settings (e.g. residential or outpatient) may impact both prescription patterns and physical activity. Therefore, Table 2 reports the sociodemographic and clinical characteristics of the sample with respect to the treatment setting. Regarding psychopathology, significant differences were found in BPRS (U = 80.977; *P* < 0.001) and BNSS (U = 83.578; *P* < 0.001) scores between outpatients and residential patients, with the latter showing higher psychopathological and negative symptoms severity. Psychosocial functioning, as measured by SLOF, was significantly better in outpatients than in residential patients (U = 106.030; *P* < 0.001). Finally, outpatients showed significantly shorter lifetime hospitalization duration than outpatients (*χ*^2^ = 235.780; *P* < 0.001). Regarding patients’ physical health, outpatients had a higher BMI (U = 103.564; *P* < 0.001) and waist circumference (U = 101.300; *P* = 0.009) than residential patients, whereas residential patients had a higher CCI (U = 89.392; *P* = 0.002). Moreover, residential patients were more likely smokers than outpatients (*χ*^2^ = 16.799; *P* < 0.001), although no differences were found between the two groups in the number of cigarettes per day smoked (U = 87.488; *P* = 0.374).

**Table 2:** Sociodemographic and clinical features of outpatients and residential patients

Variables	Outpatients *N* = 307 (49.5%)	Residential patients *N* = 313 (50.5%)	*χ*^2^/U	Effect size	*P* value^a^
Sex, *N* (%)					
Male	202 (65.8%)	220 (70.3%)	1.437	0.048	0.231
Age (mean, SD)	41.7 (9.2)	41.0 (9.7)	97.110	0.074	0.423
Marital status, *N* (%)					
Single	263 (85.7%)	271 86.9%)	11.468	0.136	**0.003**
Married or cohabiting	30 (9.8%)	13 (4.2%)			
Divorced or widowed	14 (4.6%)	28 (9.0%)			
Education (mean, SD)	11.9 (3.0)	11.5 (3.2)	98.917	0.129	0.115
Working status, *N* (%)					
Working	90 (29.3%)	38 (12.2%)	31.469	0.226	**<0.001**
Studying	21 (6.8%)	14 (4.5%)			
Not working/studying	196 (63.8%)	260 (83.3%)			
BMI (mean, SD)	28.6 (6.0)	26.9 (4.9)	103.564	0.310	**<0.001**
Waist circumference (mean, SD)	103.5 (22.2)	99.5 (15.9)	101.300	0.207	**0.009**
Smokers, *N* (%)	142 (46.4%)	196 (62.8%)	16.799	0.165	**<0.001**
Smoking (cig/day) (mean, SD)	16.8 (9.5)	16.9 (8.1)	87.488	0.011	0.374
CCI (mean, SD)	0.6 (1.1)	0.9 (1.3)	89.392	0.249	**0.002**
Illnessduration (mean, SD)	18.1 (9.4)	18.3 (9.6)	95.082	0.021	0.859
Lifetime duration of psychiatric hospitalizations, *N* (%)					
<1 year	240 (78.2%)	53 (17.0%)	235.780	0.617	**<0.001**
1–5 years	42 (13.7%)	122 (39.1%)			
>5 years	25 (8.1%)	137 (43.9%)			
BPRS (mean, SD)	42.6 (11.9)	51.0 (16.2)	80.977	0.591	**<0.001**
BNSS (mean, SD)	19.3 (13.9)	26.3 (16.6)	83.578	0.457	**<0.001**
SLOF (mean, SD)	183.2 (18.6)	174.3 (22.6)	106.030	0.430	**<0.001**
WHODAS 2.0 (mean, SD)	13.4 (9.7)	12.5 (8.7)	97.365	0.098	0.386

a Chi-square test for categorical variables, Mann–Whitney-U test for continuous variables.

BPRS, Brief Psychiatric Rating Scale; BNSS, Brief Negative Symptom Scale; CCI, Charlson Comorbidity Index; N, number; SLOF, Specific Levels of Functioning Scale; WHODAS 2.0, WHO Disability Assessment Schedule.

### Psychotropic drug prescription

Table 3 shows the pattern of prescription in the whole sample and the differences between outpatients and residential patients. Overall, 607 patients (97.9%) in the whole sample had a prescription of at least one antipsychotic, with second-generation antipsychotics (SGAs) being the most prescribed ones (69.8%). No difference was found in the overall prescription of antipsychotics between outpatients and residential patients, whereas significant differences were found for different subclasses of antipsychotics, with outpatients being more frequently prescribed SGAs and residential patients more frequently prescribed first-generation antipsychotics (FGAs). Moreover, residential patients were significantly more likely to be on APP (number of drugs prescribed) and receive clozapine than outpatients. The second most prescribed psychotropic drug category in the whole sample was benzodiazepines (53.7%), with residential patients being the more prescribed group. A lower percentage of patients in the whole sample were prescribed antidepressants (27.6%) or mood stabilizers (25.8%). Residential patients more frequently received mood stabilizers than outpatients, while no difference was found between the two groups of patients with respect to antidepressants.

**Table 3:** Pattern of prescription in the whole sample and differences between outpatients and residential patients

Drug category	N of patients receiving any drugs from each class (%)			*P* value^a^	Mean N of drugs from each class (SD)			*P* value^b^	N of drugs (range)
	Total sample *N* = 620	Outpatients *N* = 307	Residential patients *N* = 313		Total sample *N* = 620	Outpatients *N* = 307	Residential patients *N* = 313		
Antipsychotics (FGAs, SGAs, or clozapine)	607 (97.9%)	301 (98.1%)	306 (97.8%)	0.806	1.6(0.8)	1.4 (0.7)	1.8 (0.9)	**<0.001**	1–7+
FGAs	233 (37.6%)	95 (30.9%)	138 (44.1%)	**<0.001**	1.2 (0.5)	1.1 (0.4)	1.2 (0.5)	**0.040**	1–4+
SGAs	433 (69.8%)	229 (74.6%)	204 (65.2%)	**0.011**	1.2 (0.5)	1.2 (0.4)	1.3 (0.5)	**0.034**	1–3
Clozapine	154 (24.8%)	48 (15.6%)	106 (33.9%)	**<0.001**	NA	NA	NA	NA	NA
Mood stabilizers	160 (25.8%)	61 (19.9%)	99 (31.6%)	**<0.001**	1.1(0.4)	1.1 (0.3)	1.2 (0.4)	0.406	1–3
Antidepressants	171 (27.6%)	90 (29.3%)	81 (25.9%)	0.338	1.1(0.3)	1.1 (0.3)	1.1 (0.3)	0.628	1–3
Benzodiazepines	333 (53.7%)	119 (38.8%)	214 (68.4%)	**<0.001**	1.2(0.5)	1.1 (0.3)	1.4 (0.6)	**<0.001**	1–4+

a Chi-square test.

b Mann–Whitney-U test.

FGAs, first-generation antipsychotics; N, number; SGAs, second-generation antipsychotics.

The different prescription patterns of APM and APP, with respect to FGAs and SGAs, are reported in Table 4. SGAs were the most prescribed drugs in monopharmacy, with 28.6% of patients who did not assume other concomitant medications, except for benzodiazepines. At least one antidepressant was prescribed with SGAs in 9% of patients, followed by a mood stabilizer (7.6%) and one antidepressant plus a mood stabilizer (4.4%). FGAs were prescribed in monopharmacy in 9.4% of patients, without accounting for concomitant benzodiazepine prescriptions (given their higher prevalent coadministration in both APM and APP regimens). FGAs were administered with a mood stabilizer in 3.2% of patients, with at least one antidepressant in 3.1% of patients, or with their combination in 1.6% of patients treated with FGAs. Of note, 10.3% of patients were prescribed a combination of FGAs and SGAs alone, whereas 5.7 and 3.6% of patients received either a mood stabilizer or at least one antidepressant with an FGA plus SGA combination, respectively. The combination of the four drug categories was present in 0.8% of patients with SSDs.

**Table 4:** Characteristics of polypharmacy among patients with schizophrenia spectrum disorders

		*N* (%^a^)	95% CI	Plus any benzodiazepine, *N* (%)	Plus any benzodiazepine, 95% CI
First-generation antipsychotic	Alone	58 (9.4%)	7.1–11.7%	27 (4.4%)	2.8–6.0%
	Mood stabilizer	20 (3.2%)	1.8–4.6%	14 (2.3%)	1.1–3.4%
	At least one antidepressant	19 (3.1%)	1.7–4.4%	12 (1.9%)	0.9–3.0%
	Antidepressant + mood stabilizer	10 (1.6%)	0.6–2.6%	7 (1.1%)	0.3–2.0%
Second-generation antipsychotic	Alone	177 (28.6%)	25.0–32.1%	62 (10.0%)	7.6–12.4%
	Mood stabilizer	47 (7.6%)	5.5–9.7%	31 (5.0%)	3.3–6.7%
	At least one antidepressant	56 (9.0%)	1.2–6.8%	31 (5.0%)	3.3–6.7%
	Antidepressant + mood stabilizer	27 (4.4%)	2.8–6.0%	19 (3.1%)	1.7–4.4%
First- plus second-generation antipsychotic combination	Alone	64 (10.3%)	7.9–12.7%	40 (6.5%)	4.5–8.4%
	Mood stabilizer	35 (5.7%)	3.8–7.5%	23 (3.7%)	2.2–5.2%
	At least one antidepressant	22 (3.6%)	2.1–5.0%	16 (2.6%)	1.3–3.8%
	Antidepressant + mood stabilizer	5 (0.8%)	0.1–1.5%	5 (0.8%)	0.1–1.5%

a The column sum is not equal to 100% because 80 patients (12.9%) did not take first- or second-generation antipsychotics but were taking clozapine.

CI, confidence intervals; N, number.

### Physical activity

The differences in physical activity levels between patients on APM, patients on APP and healthy controls are reported in Table 5. Out of 316 patients on APM and 291 patients on APP, 73 and 57 wore wearable accelerometers, respectively. Patients on APP wore wearable accelerometers for a shorter time than patients on APM and controls. Patients spent a significantly higher amount of time (minutes per day) being sedentary compared to controls. Among patients, those on APP were less sedentary than those on APM, although no significant differences were observed. The amount of time spent in light, moderate or vigorous physical activity was significantly different between patients and controls, with the latter performing more daily physical activity in each category of physical activity intensity. No significant differences in different physical activity intensity categories were observed between patients on APP and APM, although a trend of reduced sedentariness and higher light physical activity was observed for patients on APP than patients on APM. Even when looking at the mean METs per day, controls have significantly higher values, whereas no difference was observed between patients on different treatment regimens.

**Table 5:** Differences in physical activity levels between patients (on antipsychotic mono- and polypharmacy) and healthy controls

		Patients on APM *N* = 73(29.9%)	Patients on APP *N* = 57(23.4%)	HC*N* = 114(46.7%)	*P* value^a^	Post-hoc tests *P* values			Post-hoc tests summary
						APP vs. APM	APM vs. HC	APP vs. HC	
Wearing time (hour/day)	Mean (SD)	22.3 (0.7)	21.7 (1.0)	22.5 (1.0)	**<0.001**	**0.002**	0.249	**<0.001**	APP<APM/HC
METs	mean (SD)	1.4 (0.2)	1.4 (0.2)	1.6 (0.2)	**<0.001**	0.993	**<0.001**	**<0.001**	APM/APP<HC
Sedentary (min/day)	mean (SD)	894.6 (150.5)	839.2 (138.6)	749.5 (134.7)	**<0.001**	0.068	**<0.001**	**<0.001**	HC<APP/APM
	mean % (SD)	66.2 (11.1)	63.9 (10.4)	54.7 (10.3)	**<0.001**	0.420	**<0.001**	**<0.001**	HC<APP/APM
Light (min/day)	mean (SD)	376.4 (116.8)	412.0 (115.1)	499.2 (113.1)	**<0.001**	0.187	**<0.001**	**<0.001**	APM/APP<HC
	mean % (SD)	28.6 (8.9)	32.0 (8.9)	37.5 (8.5)	**<0.001**	0.071	**<0.001**	**<0.001**	APM/APP<HC
Moderate (min/day)	mean (SD)	67.3 (62.5)	52.3 (50.1)	100.1 (55.1)	**<0.001**	0.291	**<0.001**	**<0.001**	APM/APP<HC
	mean % (SD)	5.1 (4.7)	4.0 (3.8)	7.5 (4.1)	**<0.001**	0.338	**<0.001**	**<0.001**	APM/APP<HC
Vigorous (min/day)	mean (SD)	0.7 (2.4)	0.4 (0.9)	3.8 (8.2)	**<0.001**	0.937	**0.002**	**0.001**	APM/APP<HC
	mean % (SD)	0.1 (0.2)	0.0 (0.1)	0.3 (0.6)	**<0.001**	0.938	**0.001**	**<0.001**	APM/APP<HC

a Kruskal–Wallis test was performed for wearing time, METs, moderate and vigorous activities. ANOVA was performed for sedentary and light activities.

APP, antipsychotic polypharmacy; APM, antipsychotic monopharmacy; HC, healthy controls; N, number; min, minutes; METs, metabolic equivalent of tasks.

**Fig. 1:**
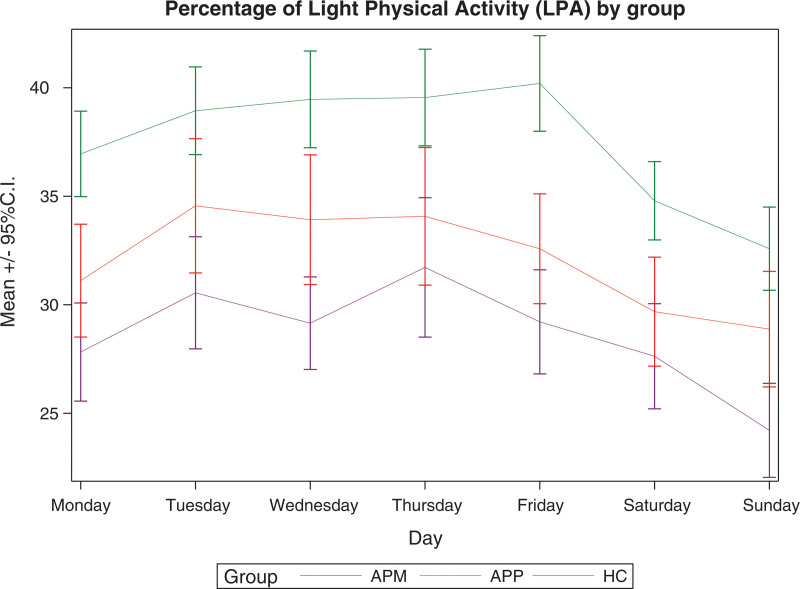
Percentage of light physical activity as assessed by accelerometers in patients on antipsychotic monopharmacy, patients on antipsychotic polypharmacy, and healthy controls during the week. APM, antipsychotic monopharmacy; APP, antipsychotic polypharmacy; CI, confidence interval; HC, healthy controls.

**Fig. 2:**
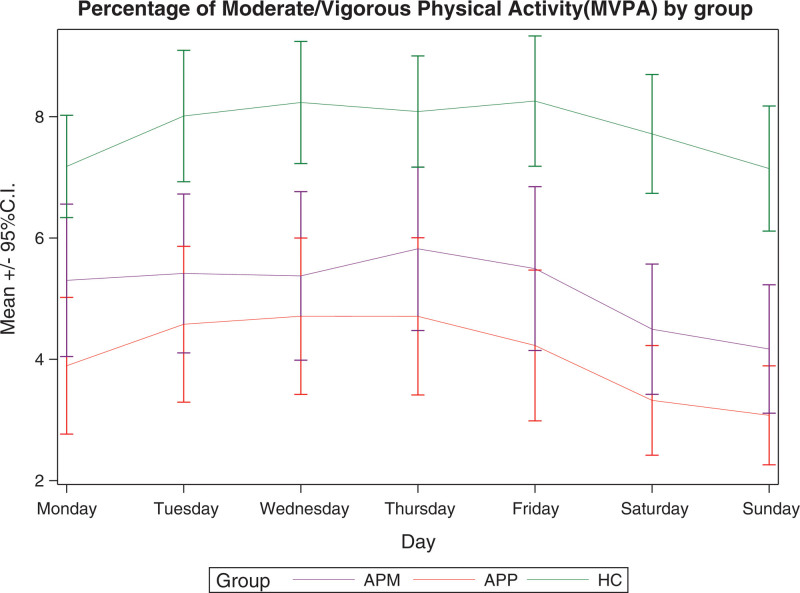
Percentage of moderate/vigorous physical activity as assessed by accelerometer in patients on antipsychotic monopharmacy, patients on antipsychotic polypharmacy, and healthy controls during the week. APM, antipsychotic monopharmacy; APP, antipsychotic polypharmacy; CI, confidence interval; HC, healthy controls.

**Fig. 3:**
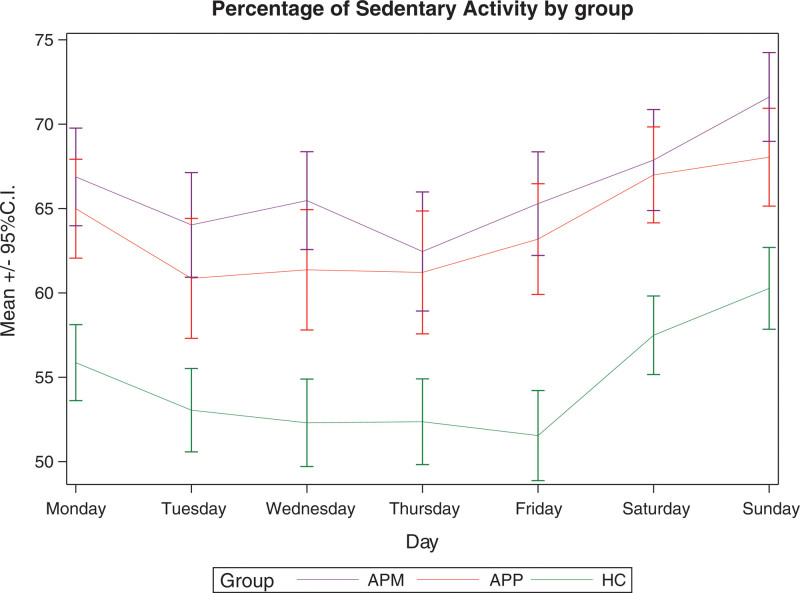
Percentage of sedentary activity as assessed by accelerometer in patients on antipsychotic monopharmacy, patients on antipsychotic polypharmacy, and healthy controls during the week. APM, antipsychotic monopharmacy; APP, antipsychotic polypharmacy; CI, confidence interval; HC, healthy controls.

Figures 1–3 show the mean daily percentages of sedentary, light and moderate/vigorous physical activity, respectively, as assessed by accelerometers in patients on APM, patients on APP and controls during the week. The trend in the curves shows that light and moderate/vigorous physical activity levels are higher on weekdays, while they decrease at weekends. Sedentary levels, on the other hand, show an opposite trend.

## Discussion

In this real-world, observational clinical study, we confirmed previous reports of a relevant rate of APP in patients with SSDs, with patients on APP showing higher disease severity than patients on APM. The relevant rate of APP was confirmed both in residential patients and outpatients, with the former being even more prone to polypharmacy than the latter. Residential patients were, as expected, more severely ill, and this may partly explain the higher rates of APP. The overall physical activity levels were significantly lower for all patients in our sample when compared to healthy controls. A trend of reduced sedentariness and higher levels of light physical activity was observed in patients on APP, which were more likely to live in psychiatric residential treatment facilities, vs. APM, possibly suggesting that rehabilitation efforts might be helpful in counteracting the generally reduced physical activity observed in patients compared to controls.

### Prescription patterns of psychotropic drugs

Antipsychotics were the most commonly prescribed pharmacological class in our sample, as expected in patients with SSDs, with SGAs being the most commonly given prescription. In terms of the characteristics associated with APM and APP, our data showed that patients on APP had greater clinical severity, as evidenced by higher BPRS and BNSS scores and a lower level of psychosocial functioning as measured by the SLOF. This may suggest that, at least in some cases, there was an attempt by clinicians to manage the increased disorder severity by using the APP. However, following the prescription of a combination of antipsychotics is controversial. The negative symptoms, which were greater in the subsample of patients on APP, may have been both determined by the disorder itself or by the use of APP (Schooler, 1994; Artaloytia *et al.*, 2006). Patients on APP were also more likely to have no partners, had a longer duration of illness and longer hospitalizations. When outpatients and residential patients were considered separately, APP was significantly more frequent in the latter, with FGAs and clozapine more frequently prescribed in this group of patients than outpatients. It is possible that the difference in prescribing patterns observed in our study between residential patients and outpatients is due, at least in part, to residential patients showing greater clinical severity than outpatients, echoing much of the differences between patients on APP and APM described above. Indeed, similarly to patients on APP, residential patients also showed higher psychopathological severity and worse psychosocial functioning, as well as longer hospitalizations (Martinelli *et al.*, 2022).

Our results are largely in line with those of previous studies investigating differences between residential patients and outpatients (Auslander *et al.*, 2001; Lee *et al.*, 2019). Noteworthy, all of these characteristics, along with the aforementioned higher clinical severity, may have led to a worse response to treatments and may have prompted the use of drug associations, as suggested by previous studies (Bolstad *et al.*, 2011; de Nijs *et al.*, 2021; Oliva *et al.*, 2022).

The second most prescribed drug class in our sample was benzodiazepines, which accounted for about 40% of prescriptions among outpatients and 70% among residential patients. Despite the wide use of this drug class in our sample, there is no consistent evidence to support the effectiveness of benzodiazepines in combination with APs on the core symptoms of SSDs. Therefore, their use should preferably be reserved by clinicians for short-term sedation of patients with acute agitation or for the treatment of AP-induced extrapyramidal effects (Dold *et al.*, 2013; Pringsheim *et al.*, 2018; Ekinci and Ekinci, 2022). The higher use of benzodiazepines among residential patients may be a consequence of greater disease chronicity, and a higher prevalence of extrapyramidal side effects related to a higher use of APP over time.

Interestingly, residential patients showed more associated comorbidities than outpatients in our sample, as evidenced by higher CCI scores. The higher comorbidity index may have a close bidirectional link with polypharmacy. Indeed, polypharmacy may lead, among others, to an increased metabolic and cardiovascular risk, whereas the presence of multimorbidity may increase the risk of lower tolerability and safety profiles of pharmacotherapy (Misawa *et al.*, 2011; Beauchemin *et al.*, 2020; Guinart and Correll, 2020; Lin, 2020).

### Physical activity and prescription of antipsychotic medications

Another aim of our study was to investigate the relationship between APM/APP and physical activity as measured by a wearable accelerometer-based biosensor. The overall physical activity levels were significantly lower for all patients in our sample compared to healthy controls, as expected, and the mean METs per day were significantly higher in controls than in patients, according to the higher levels of physical activity. The distribution of physical activity intensity among patients with SSDs, regardless of the prescribed antipsychotic therapy regimen, and controls in our sample was in line with previous evidence, which showed a pattern of less moderate physical activity and even less vigorous physical activity compared to healthy controls (Stubbs *et al.*, 2016). Several previous studies have also evaluated physical activity in residential patients and outpatients, reporting low levels of physical activity in both groups and underlining the importance of implementing physical activity in both treatment settings. More in detail, studies conducted on large samples of residential patients found that 45% of them were inactive, not even taking part in household chores at their psychiatric residential treatment facilities (de Girolamo *et al.*, 2005), and this finding was also confirmed by a more recent longitudinal study (de Girolamo *et al.*, 2014). Similarly, outpatients showed higher levels of sedentary activity and lower levels of moderate and vigorous physical activity when compared to healthy age- and sex-matched controls (Soundy *et al.*, 2013). Interestingly, we found a trend towards less sedentariness and a higher level of light physical activity in patients on APP compared to patients on APM. Given that patients on APP were more likely to live in psychiatric residential treatment facilities than those on APM, and because most psychiatric residential treatment facilities have a 24-h staff cover, it is possible that residential patients exhibited a trend of higher levels of light physical activity because they were more regularly stimulated by treating staff, as is also evident from previous studies (Mc Ardle *et al.*, 2021). Furthermore, when physical health indices were considered, residential patients showed significantly lower BMI and waist circumference than outpatients, supporting our hypothesis of a ‘compensatory’ contribution of rehabilitation programs in psychiatric residential treatment facilities, although it should also be considered that residential patients are usually subjected to a more supervised diet. Another explanation may be that patients on APP usually present more extrapyramidal side effects, such as akathisia (Pringsheim *et al.*, 2018), and this may lead to persistent higher motor activity (Poyurovsky *et al.*, 2000; Pieters *et al.*, 2021).

Because the prescription of benzodiazepines was relatively high in our sample, it is interesting to briefly consider the relationship between this class of drugs and physical activity, even if it was not part of our primary objectives. Benzodiazepines use is known to be associated with sedation, poorer physical function and limitations in activities of daily living, as well as an increased risk of falls especially in the elderly, which together may lead to a reduction in daily physical activity (Gray *et al.*, 2002; Donnelly *et al.*, 2017; Wouters *et al.*, 2020). The considerable use of benzodiazepines in the patients with SSDs in our sample may have further contributed to the lower physical activity levels observed in patients compared to healthy controls. Therefore, special attention must be paid to the management of benzodiazepine treatment in this complex patient population.

### Strengths and limitations

Our study has some strengths but also limitations. Major strengths are the real-world design of this study, with the inclusion of both residential patients and outpatients, allowing an adequate snapshot of prescribing patterns of antipsychotics in Italy. Another major strength is the inclusion of a healthy control group, which allowed a useful comparison with the patient groups. Finally, the use of objective methods to assess physical activity instead of traditional self-reports, which are subject to reporting bias. Nevertheless, the current lack of standardized procedures for actigraphy measurements may have led to overestimates, limiting comparisons between different studies. We cannot rule out the negative effect of the restrictions imposed to contain the severe acute respiratory syndrome-coronaVirus 2 (SARS-CoV-2) pandemic, during which the recruitment took place, on the measured physical activity of outpatients and healthy controls. Other limitations included the lack of data about the individual molecule and long-acting antipsychotic prescriptions and prescribed drug doses. We did not apply a formal correction for multiple testing, as we started our analyses from a clear preplanned hypothesis with a clinical exploratory aim (Amrhein *et al.*, 2019).

### Conclusion

Overall, our study found high rates of APP in both patients living in psychiatric residential treatment facilities and outpatients diagnosed with an SSD, with a relatively higher prevalence of APP among residential patients, who were also those with greater clinical severity. Deviations from international prescribing guidelines may sometimes be justified by the need to control severe and residual symptomatology but may lead to worse physical health outcomes. Concerning physical activity, this was lower for all patients in our sample when compared to healthy controls, regardless of the antipsychotic treatment regimen prescribed to patients. However, it is possible that rehabilitation efforts in psychiatric residential treatment facilities led to trends of reduced sedentariness and higher light physical activity levels in residential patients than in outpatients, even though the former were more severely ill and polymedicated with antipsychotics. Our findings may have important clinical and public health implications, as they indicate the importance of implementing awareness programs aiming at reducing APP, when possible, to limit the occurrence of physical health problems, and active rehabilitation interventions to encourage physical activity among patients with SSDs.

## Acknowledgements

The authors thank all members of the DiAPAson Consortium who actively worked to make this project possible: Norbedo Alessandro and Paolo Peressutti (Department of Mental Health, ASUGI Trieste), Daniela di Cosimo (Fatebenefratelli di San Colombano al Lambro), Maria Concetta Miranda e Caterina D’anna (Department of Mental Health, ASL Napoli 2 Nord), Livia Fussi e Antonella Di Gregorio (Department of Mental Health, ASST Melegnano e della Martesana), Marzia De Santis (Department of Mental Health, ASL Roma1), Chiara Cibra e Elisabetta Pionetti (Department of Mental Health, ASST Lodi), Nicola Necchini e Valentina Regina (Psychiatric Clinic, University of Brescia), Cosima Calini e Anna Auxilia (Psychiatric Clinic, University of Milan-Bicocca), Annalisa Maurizi e Alessandro Bellotta (Comunità Passaggi, Oricola), Angelo Brega e Pasquale Di Prisco (Cooperativa “L’Incontro”, Castelfranco Veneto), Romina Ferretti (Department of Mental Health, ASL di Teramo), Giulio D’Anna e Lorenzo Tatini (Department of Mental Health, USL Toscana Centro), Michele Tosato (Department of Mental Health, ASL3 Genovese), Vera Abbiati e Silvia Bottura (Psychiatric Clinic, University of Pavia), Giorgio Gallino e Anna Myska (Department of Mental Health, ASL Città di Torino), Giuseppina Paulillo e Emanuela Leuci (Department of Mental Health, ASL di Bari). An updated list of participating sites can be found at https://www.diapason-study.eu.

The DiAPASon (DAily time use, Physical Activity, quality of care and interpersonal relationships in patients with Schizophrenia spectrum disorders) Consortium includes: Arturo Rippa (Department of Mental Health, ASUGI Trieste), Roberto Placenti (Fatebenefratelli di San Colombano al Lambro), Vittorio Di Michele (Department of Mental Health, ASL Pescara), Maria Gabriella Foia (Department of Mental Health, ASL Napoli 2 Nord), Federico Durbano (Department of Mental Health, ASST Melegnano e della Martesana), Tommaso Achille Poliseno (Department of Mental Health, ASL Roma1), Giancarlo Cerveri (Department of Mental Health, ASST Lodi), Michele Facchi (Psychiatric Clinic, University of Brescia), Rodolfo Pessina (Psychiatric Clinic, University of Milan-Bicocca), Filippo M. Jacoponi (Comunità Passaggi, Oricola), Roberto De Marchi (Cooperativa “Il Girasole”, Treviso), Patricia Giosuè (Department of Mental Health, ASL di Teramo), Andrea Baroncelli (Department of Mental Health, USL Toscana Centro), Lucio Ghio (Department of Mental Health, ASL3 Genovese), Filippo Besana (Psychiatric Clinic, University of Pavia), Federico Facchini (Department of Mental Health, ASL Città di Torino), Lorenzo Pelizza (Department of Mental Health, AUSL di Parma), Valeria Latorre (Department of Mental Health, ASL di Bari).

The DiAPASon project is entirely funded by the Italian Ministry of Health (Bando per la Ricerca Finalizzata 2018: RF-2018-12365514). The Italian Ministry of Health organized the peer review of the study protocol to grant approval. The Italian Ministry of Health has no role in the analyses and interpretation of study findings.

The dataset referring to this manuscript is published with restricted access on the Zenodo platform at this link: https://doi.org/10.5281/zenodo.6651539.

### Conflicts of interest

A. Serretti is or has been a consultant/speaker for Abbott, Abbvie, Angelini, AstraZeneca, Clinical Data, Boehringer, Bristol-Myers Squibb, Eli Lilly, GlaxoSmithKline, Innovapharma, Italfarmaco, Janssen, Lundbeck, Naurex, Pfizer, Polifarma, Sanofi, Servier, and Taliaz. The other authors declare no conflicts of interest.

## References

[R1] American Psychiatric Association. (2013). Diagnostic and statistical manual of mental disorders (5th ed.).

[R2] Amrhein V Greenland S McShane B (2019). Scientists rise up against statistical significance. Nature 567:305–307. 30894741 10.1038/d41586-019-00857-9

[R3] Artaloytia JF Arango C Lahti A Sanz J Pascual A Cubero P. (2006). Negative signs and symptoms secondary to antipsychotics: a double-blind, randomized trial of a single dose of placebo, haloperidol, and risperidone in healthy volunteers. Am J Psychiatry 163:488–493. 16513871 10.1176/appi.ajp.163.3.488

[R4] Auslander LA Lindamer LL Delapena J Harless K Polichar D Patterson TL. (2001). A comparison of community-dwelling older schizophrenia patients by residential status. Acta Psychiatr Scand 103:380–386. 11380308 10.1034/j.1600-0447.2001.00262.x

[R5] Beauchemin M Geguchadze R Guntur AR Nevola K Le PT Barlow D. (2020). Exploring mechanisms of increased cardiovascular disease risk with antipsychotic medications: risperidone alters the cardiac proteomic signature in mice. Pharmacol Res 152:104589. 31874253 10.1016/j.phrs.2019.104589 PMC7060507

[R6] Bellantuono C Reggi V Tognoni G Muscettola G. (1981). Comparison of practice in hospitals and community mental health centers. In: Epidemiological impact of psychotropic drugs. Elsevier. pp. 171–181.

[R7] Bolstad A Andreassen OA Røssberg JI Agartz I Melle I Tanum L (2011). Previous hospital admissions and disease severity predict the use of antipsychotic combination treatment in patients with schizophrenia. BMC Psychiatry 11:126. 21812996 10.1186/1471-244X-11-126 PMC3160878

[R8] Caliskan AM Karaaslan M Inanli I Caliskan S Arslan M Esra Cicek I Eren I (2021). The effects of adding long-acting injectable antipsychotic drugs to clozapine on relapse and hospitalization in patients with treatment-resistant schizophrenia: a mirror-image retrospective study. Int Clin Psychopharmacol 36:30–33. 33044315 10.1097/YIC.0000000000000336

[R9] Carton L Cottencin O Lapeyre-Mestre M Geoffroy PA Favre J Simon N. (2015). Off-label prescribing of antipsychotics in adults, children and elderly individuals: a systematic review of recent prescription trends. Curr Pharm Des 21:3280–3297. 26088115 10.2174/1381612821666150619092903

[R10] Charlson ME Pompei P Ales KL MacKenzie CR (1987). A new method of classifying prognostic comorbidity in longitudinal studies: development and validation. J Chronic Dis 40:373–383. 3558716 10.1016/0021-9681(87)90171-8

[R11] Chen LJ Steptoe A Chung MS Ku PW (2016). Association between actigraphy-derived physical activity and cognitive performance in patients with schizophrenia. Psychol Med 46:2375–2384. 27283122 10.1017/S0033291716000921

[R12] Cooper SJ Reynolds GP Barnes T England E Haddad PM Heald A; With expert co-authors (in alphabetical order) (2016). BAP guidelines on the management of weight gain, metabolic disturbances and cardiovascular risk associated with psychosis and antipsychotic drug treatment. J Psychopharmacol 30:717–748. 27147592 10.1177/0269881116645254

[R13] Correll CU Rummel-Kluge C Corves C Kane JM Leucht S (2009). Antipsychotic combinations vs monotherapy in schizophrenia: a meta-analysis of randomized controlled trials. Schizophr Bull 35:443–457. 18417466 10.1093/schbul/sbn018 PMC2659301

[R14] De Berardis D Vellante F Fornaro M Orsolini L Valchera A Baroni G. (2020). Rapid improvement of obsessive-compulsive disorder associated with schizophrenia with cariprazine add-on in a subject under paliperidone long-acting injection: a case report. Int Clin Psychopharmacol 35:113–118. 32004167 10.1097/YIC.0000000000000284

[R15] de Girolamo G Williams P Cappiello V (1987). Psychotropic drug utilization and audit in two Italian psychiatric services. Psychol Med 17:989–997. 2893408 10.1017/s0033291700000805

[R16] de Girolamo G Picardi A Santone G Falloon I Morosini P Fioritti A Micciolo R; PROGRES Group. (2005). The severely mentally ill in residential facilities: a national survey in Italy. Psychol Med 35:421–431. 15841877 10.1017/s0033291704003502

[R17] de Girolamo G Candini V Buizza C Ferrari C Boero ME Giobbio GM. (2014). Is psychiatric residential facility discharge possible and predictable? A multivariate analytical approach applied to a prospective study in Italy. Soc Psychiatry Psychiatr Epidemiol 49:157–167. 23712514 10.1007/s00127-013-0705-z

[R18] de Girolamo G Rocchetti M Benzi IMA Agosta S Casiraghi L Ferrari C. (2020). DAily time use, Physical Activity, quality of care and interpersonal relationships in patients with Schizophrenia spectrum disorders (DiAPASon): an Italian multicentre study. BMC Psychiatry 20:287. 32513140 10.1186/s12888-020-02588-y PMC7278132

[R19] de Nijs J Burger TJ Janssen RJ Kia SM van Opstal DPJ de Koning MB; GROUP investigators. (2021). Individualized prediction of three- and six-year outcomes of psychosis in a longitudinal multicenter study: a machine learning approach. NPJ Schizophr 7:34. 34215752 10.1038/s41537-021-00162-3 PMC8253813

[R20] Dold M Li C Gillies D Leucht S (2013). Benzodiazepine augmentation of antipsychotic drugs in schizophrenia: a meta-analysis and Cochrane review of randomized controlled trials. Eur Neuropsychopharmacol 23:1023–1033. 23602690 10.1016/j.euroneuro.2013.03.001

[R21] Donnelly K Bracchi R Hewitt J Routledge PA Carter B (2017). Benzodiazepines, Z-drugs and the risk of hip fracture: a systematic review and meta-analysis. PLoS One 12:e0174730. 28448593 10.1371/journal.pone.0174730 PMC5407557

[R22] Ekinci O Ekinci A (2022). Short-term, but not long-term, beneficial effects of concomitant benzodiazepine use on clinical course in patients with schizophrenia. Int Clin Psychopharmacol 37:143–150. 35045532 10.1097/YIC.0000000000000392

[R23] Esteves PS Mota D Cerejeira J Mendes F. (2015). Low doses of adjunctive aripiprazole as treatment for antipsychotic-induced hyperprolactinemia: a literature review. Eur Psychiatry 30:1–1.25169445

[R24] Federici S Meloni F Mancini A Lauriola M Olivetti Belardinelli M (2009). World health organisation disability assessment schedule II: contribution to the Italian validation. Disabil Rehabil 31:553–564. 19191060 10.1080/09638280802240498

[R25] Firth J Stubbs B Rosenbaum S Vancampfort D Malchow B Schuch F. (2017). Aerobic exercise improves cognitive functioning in people with schizophrenia: a systematic review and meta-analysis. Schizophr Bull 43:546–556. 27521348 10.1093/schbul/sbw115 PMC5464163

[R26] Firth J Stubbs B Vancampfort D Schuch FB Rosenbaum S Ward PB. (2018). The validity and value of self-reported physical activity and accelerometry in people with schizophrenia: a population-scale study of the UK biobank. Schizophr Bull 44:1293–1300. 29069474 10.1093/schbul/sbx149 PMC6192495

[R27] Fisher MD Reilly K Isenberg K Villa KF (2014). Antipsychotic patterns of use in patients with schizophrenia: polypharmacy versus monotherapy. BMC Psychiatry 14:341. 25433495 10.1186/s12888-014-0341-5 PMC4264319

[R28] Gallego JA Bonetti J Zhang J Kane JM Correll CU (2012). Prevalence and correlates of antipsychotic polypharmacy: a systematic review and meta-regression of global and regional trends from the 1970s to 2009. Schizophr Res 138:18–28. 22534420 10.1016/j.schres.2012.03.018 PMC3382997

[R29] Galling B Roldán A Hagi K Rietschel L Walyzada F Zheng W. (2017). Antipsychotic augmentation vs. monotherapy in schizophrenia: systematic review, meta-analysis and meta-regression analysis. World Psychiatry 16:77–89. 28127934 10.1002/wps.20387 PMC5269492

[R30] Gold LH (2014). DSM-5 and the assessment of functioning: the World Health Organization disability assessment schedule 2.0 (WHODAS 2.0). J Am Acad Psychiatry Law 42:173–181. 24986344

[R31] Gray SL LaCroix AZ Blough D Wagner EH Koepsell TD Buchner D (2002). Is the use of benzodiazepines associated with incident disability? J Am Geriatr Soc 50:1012–1018. 12110059 10.1046/j.1532-5415.2002.50254.x PMC4776743

[R32] Guinart D Correll CU (2020). Antipsychotic polypharmacy in schizophrenia: why not? J Clin Psychiatry 81:1101. 10.4088/JCP.19ac13118 32369690

[R33] Gündoğmuş İ Aydin MB Öz S Taşçi AB Uzun Ö (2021). Clinical and demographic factors associated with early relapse in patients with schizophrenia: a naturalistic observation study. Int Clin Psychopharmacol 36:288–295. 34417787 10.1097/YIC.0000000000000377

[R34] Hildebrand M VAN Hees VT Hansen BH Ekelund U (2014). Age group comparability of raw accelerometer output from wrist- and hip-worn monitors. Med Sci Sports Exerc 46:1816–1824. 24887173 10.1249/MSS.0000000000000289

[R35] Kodama S Saito K Tanaka S Maki M Yachi Y Asumi M. (2009). Cardiorespiratory fitness as a quantitative predictor of all-cause mortality and cardiovascular events in healthy men and women: a meta-analysis. JAMA 301:2024–2035. 19454641 10.1001/jama.2009.681

[R36] Lähteenvuo M Tiihonen J (2021). Antipsychotic polypharmacy for the management of schizophrenia: evidence and recommendations. Drugs 81:1273–1284. 34196945 10.1007/s40265-021-01556-4 PMC8318953

[R37] Lee EE Martin AS Kaufmann CN Liu J Kangas J Daly RE. (2019). Comparison of schizophrenia outpatients in residential care facilities with those living with someone: study of mental and physical health, cognitive functioning, and biomarkers of aging. Psychiatry Res 275:162–168. 30925304 10.1016/j.psychres.2019.02.067 PMC6504557

[R38] Lin SK (2020). Antipsychotic polypharmacy: a dirty little secret or a fashion? Int J Neuropsychopharmacol 23:125–131. 31867671 10.1093/ijnp/pyz068 PMC7093996

[R39] Lippi M Fanelli G Fabbri C De Ronchi D Serretti A (2022). The dilemma of polypharmacy in psychosis: is it worth combining partial and full dopamine modulation? Int Clin Psychopharmacol. [Ahead of print]. 10.1097/YIC.0000000000000417 PMC9521590 35815937

[R40] Martinelli A Iozzino L Pozzan T Cristofalo D Bonetto C Ruggeri M (2022). Performance and effectiveness of step progressive care pathways within mental health supported accommodation services in Italy. Soc Psychiatry Psychiatr Epidemiol 57:939–952. 35041014 10.1007/s00127-021-02128-3

[R41] Mc Ardle R Sverdrup K Del Din S Lord S Kerse N Rochester L Taylor L (2021). Quantifying physical activity in aged residential care facilities: a structured review. Ageing Res Rev 67:101298. 33592308 10.1016/j.arr.2021.101298

[R42] Migueles JH Rowlands AV Huber F Sabia S van Hees VT (2019). GGIR: a research community–driven open source R package for generating physical activity and sleep outcomes from multi-day raw accelerometer data. J Meas Phys Behav 2:188–196.

[R43] Misawa F Shimizu K Fujii Y Miyata R Koshiishi F Kobayashi M. (2011). Is antipsychotic polypharmacy associated with metabolic syndrome even after adjustment for lifestyle effects?: a cross-sectional study. BMC Psychiatry 11:118. 21791046 10.1186/1471-244X-11-118 PMC3155482

[R44] Mittal VA Vargas T Osborne KJ Dean D Gupta T Ristanovic I. (2017). Exercise treatments for psychosis: a review. Curr Treat Options Psychiatry 4:152–166. 29034144 10.1007/s40501-017-0112-2 PMC5636011

[R45] Montemagni C Rocca P Mucci A Galderisi S Maj M. (2015). Italian version of the ‘Specific Level of Functioning’. J Psychopathol 21:287–296.

[R46] Morosini PL Casacchia M. (1995). Italian translation of the Brief Psychiatric Rating Scale, version 4.0 expanded (BPRS 4.0). Rivista di Riabilitazione Psichiatrica e Psicosociale 3:199–228.

[R47] Mucci A Galderisi S Merlotti E Rossi A Rocca P Bucci P; Italian Network for Research on Psychoses. (2015). The brief negative symptom scale (BNSS): independent validation in a large sample of Italian patients with schizophrenia. Eur Psychiatry 30:641–647. 25758156 10.1016/j.eurpsy.2015.01.014

[R48] Muscettola G Bollini P Pampallona S (1991). Pattern of neuroleptic drug use in Italian mental health services. DICP 25:296–301. 1674184 10.1177/106002809102500315

[R49] Muscettola G Casiello M Bolline P Sebastiani G Pampallona S Tognoni G (1987). Pattern of therapeutic intervention and role of psychiatric settings: a survey in two regions of Italy. Acta Psychiatr Scand 75:55–61. 3577841 10.1111/j.1600-0447.1987.tb02751.x

[R50] Oliva V Fanelli G Kasper S Zohar J Souery D Montgomery S. (2022). Social withdrawal as a trans-diagnostic predictor of short-term remission: a meta-analysis of five clinical cohorts. Int Clin Psychopharmacol 37:38–45. 34855649 10.1097/YIC.0000000000000384

[R51] Overall JE Gorham DR. (1962). The brief psychiatric rating scale. Psychol Rep 10:799–812.

[R52] Pieters LE Deenik J Tenback DE van Oort J van Harten PN (2021). Exploring the relationship between movement disorders and physical activity in patients with schizophrenia: an actigraphy study. Schizophr Bull 47:906–914. 33764476 10.1093/schbul/sbab028 PMC8266591

[R53] Poyurovsky M Nave R Epstein R Tzischinsky O Schneidman M Barnes TR. (2000). Actigraphic monitoring (actigraphy) of circadian locomotor activity in schizophrenic patients with acute neuroleptic-induced akathisia. Eur Neuropsychopharmacol 10:171–176. 10793319 10.1016/s0924-977x(00)00063-8

[R54] Prince SA Adamo KB Hamel ME Hardt J Connor Gorber S Tremblay M (2008). A comparison of direct versus self-report measures for assessing physical activity in adults: a systematic review. Int J Behav Nutr Phys Act 5:56. 18990237 10.1186/1479-5868-5-56 PMC2588639

[R55] Pringsheim T Gardner D Addington D Martino D Morgante F Ricciardi L. (2018). The assessment and treatment of antipsychotic-induced akathisia. Can J Psychiatry 63:719–729. 29685069 10.1177/0706743718760288 PMC6299189

[R56] Schooler NR (1994). Deficit symptoms in schizophrenia: negative symptoms versus neuroleptic-induced deficits. Acta Psychiatr Scand Suppl 380:21–26. 7914043 10.1111/j.1600-0447.1994.tb05827.x

[R57] Simon GE Stewart C Yarborough BJ Lynch F Coleman KJ Beck A. (2018). Mortality rates after the first diagnosis of psychotic disorder in adolescents and young adults. JAMA Psychiatry 75:254–260. 29387876 10.1001/jamapsychiatry.2017.4437 PMC5885951

[R58] Soundy A Wampers M Probst M De Hert M Stubbs B Vancampfort D. (2013). Physical activity and sedentary behaviour in outpatients with schizophrenia: a systematic review and meta-analysis. Int J Ther Rehabil 20:588–595.

[R59] Strauss GP Keller WR Buchanan RW Gold JM Fischer BA McMahon RP. (2012). Next-generation negative symptom assessment for clinical trials: validation of the Brief Negative Symptom Scale. Schizophr Res 142:88–92. 23127378 10.1016/j.schres.2012.10.012 PMC3502630

[R60] Stubbs B Firth J Berry A Schuch FB Rosenbaum S Gaughran F. (2016). How much physical activity do people with schizophrenia engage in? A systematic review, comparative meta-analysis and meta-regression. Schizophr Res 176:431–440. 27261419 10.1016/j.schres.2016.05.017

[R61] Tomasi R de Girolamo G Santone G Picardi A Micciolo R Semisa D Fava S; PROGRES Group. (2006). The prescription of psychotropic drugs in psychiatric residential facilities: a national survey in Italy. Acta Psychiatr Scand 113:212–223. 16466405 10.1111/j.1600-0447.2005.00657.x

[R62] van Hees VT Gorzelniak L Dean León EC Eder M Pias M Taherian S. (2013). Separating movement and gravity components in an acceleration signal and implications for the assessment of human daily physical activity. PLoS One 8:e61691. 23626718 10.1371/journal.pone.0061691 PMC3634007

[R63] Vancampfort D Probst M Daenen A Damme TV De Hert M Rosenbaum S Bruyninckx D (2016). Impact of antipsychotic medication on physical activity and physical fitness in adolescents: an exploratory study. Psychiatry Res 242:192–197. 27288738 10.1016/j.psychres.2016.05.042

[R64] Vancampfort D Firth J Schuch FB Rosenbaum S Mugisha J Hallgren M. (2017). Sedentary behavior and physical activity levels in people with schizophrenia, bipolar disorder and major depressive disorder: a global systematic review and meta-analysis. World Psychiatry 16:308–315. 28941119 10.1002/wps.20458 PMC5608847

[R65] Walther S Stegmayer K Horn H Razavi N Müller TJ Strik W (2014). Physical activity in schizophrenia is higher in the first episode than in subsequent ones. Front Psychiatry 5:191. 25601842 10.3389/fpsyt.2014.00191 PMC4283447

[R66] Wee ZY Yong SWL Chew QH Guan C Lee TS Sim K (2019). Actigraphy studies and clinical and biobehavioural correlates in schizophrenia: a systematic review. J Neural Transm (Vienna) 126:531–558. 30888511 10.1007/s00702-019-01993-2

[R67] Wouters H Hilmer SN Twisk J Teichert M Van Der Meer HG Van Hout HPJ Taxis K (2020). Drug burden index and cognitive and physical function in aged care residents: a longitudinal study. J Am Med Dir Assoc 21:1086–1092.e1. 32736845 10.1016/j.jamda.2020.05.037

